# Is there a role for doravirine in African HIV treatment programmes? A large observational resistance study in South Africa

**DOI:** 10.1002/jia2.25706

**Published:** 2021-05-04

**Authors:** Kim Steegen, Michelle Moorhouse, Annemarie MJ Wensing, Willem DF Venter, Lucia Hans

**Affiliations:** ^1^ Department of Molecular Medicine and Haematology National Health Laboratory Services Johannesburg South Africa; ^2^ Department of Molecular Medicine and Haematology University of the Witwatersrand Johannesburg South Africa; ^3^ Ezintsha, Faculty of Health Sciences University of the Witwatersrand Johannesburg South Africa; ^4^ Translational Virology Department of Medical Microbiology University Medical Center Utrecht Utrecht The Netherlands; ^5^ Global Medical Affairs ViiV HealthCare Johannesburg South Africa

**Keywords:** HIV, drug resistance, antiretroviral treatment, doravirine, subtype C, South Africa

## Abstract

**Introduction:**

Dolutegravir has replaced efavirenz in most low‐ and middle‐income countries, due to better tolerability and formidable resistance profile, but dolutegravir side effects suggest alternatives are needed. We evaluated doravirine resistance in South Africa as a first step to assess whether doravirine may replace dolutegravir.

**Methods:**

A retrospective dataset was analysed for predicted doravirine susceptibility, including sequences obtained from three patient groups. First, data from 277 patients initiating antiretroviral treatment (ART) were collected between February 2013 and October 2014 as part of a national survey. Second, data from 788 patients experiencing NNRTI‐based ART failure were obtained between February 2013 and October 2014 as part of a national survey. Third, data derived from 584 patients who had genotypic drug resistance testing requested after NNRT‐based failure as part of individual patient management between January 2016 and December 2019. *Pol* sequences were generated using validated population‐based in‐house genotyping and submitted to Stanford HIVdb v8.9.

**Results and discussion:**

Less than 5% of patients initiating ART presented with genotypic doravirine resistance, whereas most patients experiencing NNRTI‐based ART failure presented with predicted intermediate (41.0%) or high‐level resistance (43.8%) to doravirine. High‐level resistance to doravirine was commonly predicted by the presence of at least three DRMs (79.7%). The predicted resistance profile to doravirine in ART‐naïve patients is promising, but less so in those experiencing failure to first‐generation NNRTIs. Accumulation of NNRTI DRMs seems to be an important factor in the poor resistance prediction for doravirine.

**Conclusions:**

Although doravirine is approved as initial therapy in patients who are ART‐naïve, it is currently recommended to obtain a genotype prior to the initiation of ART. Clinical studies are needed to ascertain whether predicted resistance profiles in ART naïve and NNRTI‐treated patients translate into poor clinical outcomes, especially in settings where genotypic resistance testing is not available.

## 1 Introduction

South Africa has the largest HIV epidemic globally with 7.5 million estimated infections and approximately 5.2 million HIV‐infected people on antiretroviral treatment (ART) making it the biggest ART programme in the world [1]. The preferred first‐line regimen in South Africa is tenofovir–lamivudine–dolutegravir (TLD), introduced in late 2019 following a WHO recommendation [2, 3]. Dolutegravir replaced efavirenz for first‐line ART in light of rising regional non‐nucleoside reverse transcriptase inhibitor (NNRTI) resistance, as well as for selected second‐line patients. In clinical studies, dolutegravir demonstrated excellent tolerability, a formidable resistance barrier [4], and provides cost benefits over efavirenz‐based regimens in generic co‐formulations in lower and middle‐income countries (LMICs) [5].

However, with wider use of dolutegravir, concerns about significant weight gain among African women treated with integrase inhibitors (InSTIs) have been raised [6, 7]. It is prudent to consider which other antiretrovirals might be suitable for first‐line ART in LMICs, taking into account the moderate to high level of pre‐treatment NNRTI resistance in this region [8]. Due to the limited availability of genotypic resistance testing these drugs would ideally have sufficient predicted efficacy avoiding the requirement of limited drug resistance testing resources.

Doravirine, a third‐generation NNRTI, may be an alternative to dolutegravir, with comparable virological suppression for doravirine compared to ritonavir‐boosted darunavir [9, 10] and efavirenz [11, 12]. *In vitro* experiments indicate a different resistance pathway, and possible clinical efficacy to isolates with several NNRTI mutations [13, 14, 15].

We assessed the level of predicted resistance to doravirine in ART‐naïve and patients experiencing NNRTI‐based ART failure in South Africa.

## 2 Materials and Methods

### 2.1 Study population

Genotypic sequence data from two sources were included for this analysis. First, a well‐characterized cohort of patients initiating ART (n = 277) [16] and patients experiencing NNRTI‐based ART failure (n = 788) [17] were obtained during a national HIV drug resistance surveillance project in South Africa. In this survey, probability proportional to size sampling was used to achieve a proportional distribution of ART‐naïve individuals. Samples were collected between February 2013 and October 2014, when efavirenz‐based ART had been the mainstay of ART regimens since 2004. Second, HIV drug resistance results obtained between January 2016 and December 2018 from patients experiencing NNRTI‐based ART failure were retrieved from the database at the accredited Charlotte Maxeke Johannesburg Academic Hospital HIV genotyping laboratory (n = 584) which receives specimens from three provinces. HIV drug resistance testing for patients failing NNRTI regimens is requested at the clinician’s discretion. Demographic and clinical data were collected from study questionnaires and laboratory request forms.

### 2.2 Pol genotyping and *in silico* analysis of sequences

*Pol* sequences were generated using validated population‐based in‐house genotyping methods, depending on the time of sampling [18, 20].

The 2019 IAS‐USA drug resistance mutation tables were used to identify HIVDR mutations [21], and the Stanford HIVdb v8.9 tool was used to interpret resistance profiles, categorized as susceptible, intermediate or high‐level resistance. Subtyping was performed using the Rega HIV subtyping tool v3.0 via Stanford (https://hivdb.stanford.edu/).

The *pol* nucleotide sequences were submitted to GenBank using Bankit (https://www.ncbi.nlm.nih.gov/WebSub/); accession numbers: KU127587‐KU128374, KT892975‐KT893251 and MW125724 ‐ MW126307.

### 2.3 Statistical analysis

Statistical analyses were performed using GraphPad QuickCalcs (http://www.graphpad.com/quickcalcs). Two‐sided Fisher’s exact tests were used to compare mutation prevalence between groups, considering a *p* < 0.05 as statistically significant.

### 2.4 Ethics statement

This study was conducted in accordance with the Declaration of Helsinki and national and institutional standards. Ethical clearance was obtained by the Research on Human Subjects (Medical) Committee at the University of the Witwatersrand (Clearance Number M181158 and M190593).

## 3 Results and Discussion

Patients initiating ART (n = 277) had a median age of 34 years and most were women (58.8%). The most common *pol* subtype was HIV‐1 subtype C (98.6%). Overall, 47 out of 277 surveyed patients presented with at least one NNRTI drug resistance mutation (DRM, Table 1). However, half (n = 23) presented with polymorphic mutations only: E138A/G (n = 17), E138A+V179D (n = 2), V179D (n = 2), Y181H (n = 1) and A98G (n = 1). For the remaining 24, the most common NNRTI mutation was K103N (n = 16) followed by Y181C (n = 6) and V106M (n = 3).

**Table 1:** Antiretroviral‐naïve patients with at least one drug resistance mutation and their predicted resistance profiles to non‐nucleoside reverse transcriptase inhibitors

Sample ID	NNRTI DRM	DOR	EFV	NVP
13ZAGFFSA007	K103N,V106M,Y181C,H221HY	HLR	HLR	HLR
13ZAGFECA020	Y188L	HLR	HLR	HLR
13ZAGFKZA014	K103N,V106M,M230L	HLR	HLR	HLR
14ZAGFKZA051	K101E,*V106I*,Y181C,G190A	HLR	HLR	HLR
13ZAGFECA018	K101H,G190S	IR	HLR	HLR
14ZAGFKZA092	K103KN,Y181YC	IR	HLR	HLR
14ZAGFKZA111	K103N,P225H	IR	HLR	HLR
14ZAGFMPA020	K103N,Y181YC	IR	HLR	HLR
13ZAGFKZA003	V106M	IR	HLR	HLR
13ZAGFFSA012	L100LI,*E138A*	IR	HLR	HLR
14ZAGFKZA076	*A98AG*,V108VI	IR	IR	IR
13ZAGFGPA021	Y181YC,H221HY	IR	IR	HLR
14ZAGFKZA079	*A98G*	IR	IR	IR
13ZAGFFSA001	K103KN	S	HLR	HLR
13ZAGFECA015	K103N	S	HLR	HLR
13ZAGFECA013	K103N	S	HLR	HLR
13ZAGFGPA049	K103N	S	HLR	HLR
13ZAGFKZA035	K103N	S	HLR	HLR
14ZAGFNWA003	K103N	S	HLR	HLR
14ZAGFKZA063	K103N	S	HLR	HLR
14ZAGFKZA057	K103N	S	HLR	HLR
14ZAGFKZA089	K103N	S	HLR	HLR
14ZAGFKZA099	K103N	S	HLR	HLR
14ZAGFGPA034	K103N,*E138A*	S	HLR	HLR
14ZAGFLPA011	Y181YC	S	IR	HLR
14ZAGFMPA025	*V179VD*	S	IR	IR
13ZAGFGPA006	*E138A,V179D*	S	S	S
14ZAGFLPA006	*E138EA,V179VDE*	S	S	S
13ZAGFGPA007	*E138A*	S	S	S
13ZAGFFSA023	*E138A*	S	S	S
13ZAGFECA010	*E138A*	S	S	S
13ZAGFKZA028	*E138A*	S	S	S
13ZAGFKZA038	*E138A*	S	S	S
14ZAGFKZA061	*E138A*	S	S	S
14ZAGFLPA004	*E138A*	S	S	S
14ZAGFKZA085	*E138A*	S	S	S
14ZAGFLPA009	*E138A*	S	S	S
14ZAGFKZA095	*E138A*	S	S	S
14ZAGFMPA031	*E138A*	S	S	S
14ZAGFWCA002	*E138A*	S	S	S
14ZAGFECA021	*E138A*	S	S	S
14ZAGFKZA070	*E138A*	S	S	S
13ZAGFFSA021	*E138EA*	S	S	S
13ZAGFGPA009	*E138EG*	S	S	S
13ZAGFFSA016	*E138EG*	S	S	S
13ZAGFGPA008	*V179VD*	S	S	S
13ZAGFFSA019	*Y181YH*	S	S	S

Polymorphic mutations are indicated in italics. DRM, drug resistance mutation; DOR, doravirine; EFV, efavirenz; HLR, high‐level resistance; IR, intermediate resistance; NNRTI, non‐nucleoside reverse transcriptase inhibitor; NVP, nevirapine; S, susceptible.

In the cohort of patients initiating ART, 4.7% (n = 13) presented with genotypic doravirine resistance compared to 9.4% (n = 26) with efavirenz/nevirapine resistance (*p* = 0.0451). Only four patients (1.4%) with doravirine resistance harboured high‐level resistance, compared to 8.3% for nevirapine and 7.6% for efavirenz. Among patients with doravirine resistance, three patients presented with a single NNRTI mutation, whereas in 10 patients, two to four NNRTI mutations were detected (Table 1). Prior undisclosed ART exposure, which could account for the detection of multiple DRMs, could not be verified in these patients.

**Figure 1:**
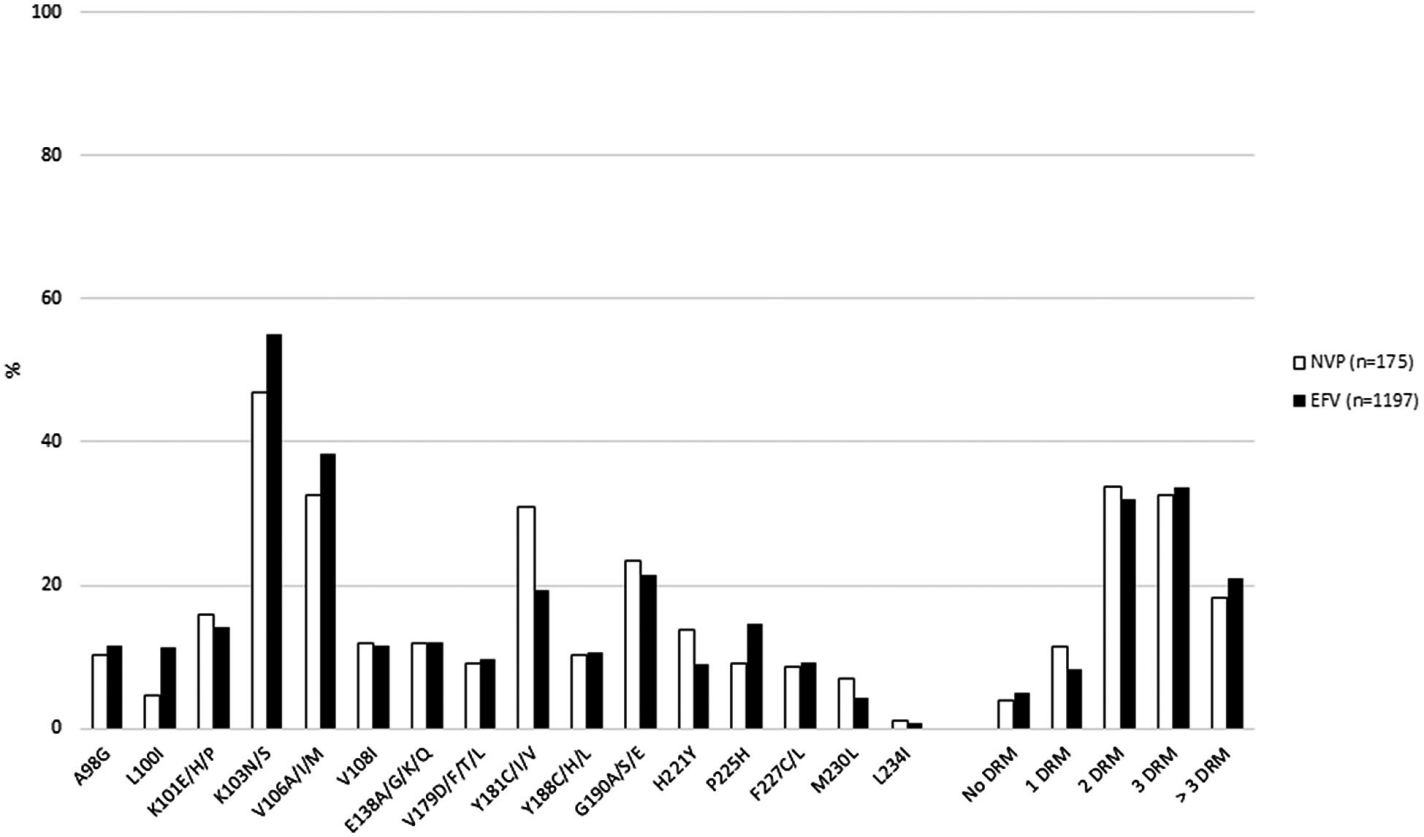
Prevalence of non‐nucleoside reverse transcriptase inhibitor (NNRTI) mutations among 1372 patients experiencing efavirenz (n = 1197) or nevirapine (n = 175) based ART failure. Exposure to efavirenz or nevirapine did not affect the prevalence of most DRMs, except for L100I, K103N/S and P225H, which were more frequently detected in efavirenz‐exposed patients (*p* = 0.0051, *p* = 0.0425 and *p* = 0.0476 respectively); whereas Y181C/I/V were more commonly detected in patients experiencing nevirapine‐based regimen failure (*p* = 0.0009). The accumulation of drug resistance mutations did not differ by treatment group. DRM, drug resistance mutation; EFV, efavirenz; NVP, nevirapine.

Patients experiencing NNRTI‐based ART failure (n = 1372) were mostly female (63.6%), had a median age of 37 years, and a median HIV viral load of 4.8 log copies/mL (IQR: 4.3 – 5.3 log copies/mL). The most common *pol* subtype was HIV‐1 subtype C (98.5%). Patients were predominantly treated with efavirenz‐based regimens (87.2%, n = 1197); duration of treatment was not available. At least one NNRTI DRM was detected in 95.1% of patients (n = 1305). The most common NNRTI DRMs were K103N/S (54.1%, n = 742), V106A/I/M (37.7%, n = 517), G190A/S/E (21.6%, n = 297) and Y181C/I/V (20.8%, n = 286). Exposure to efavirenz or nevirapine did not affect the prevalence of most DRMs, except for L100I, K103N/S and P225H, which were more frequently detected in efavirenz‐exposed patients (*p* = 0.0051, *p* = 0.0425 and *p* = 0.0476 respectively); whereas Y181C/I/V were more commonly detected in patients experiencing nevirapine‐based regimen failure (*p* = 0.0009). Figure 1 depicts the prevalence of NNRTI mutations across the different treatment groups.

Only a minority of patients retained full susceptibility to efavirenz and nevirapine (5.7%, n = 67) compared to 15.2% (n = 209) of patients who remained fully susceptible to doravirine (*p* < 0.0001). However, 41.0% and 43.8% of patients were predicted to harbour intermediate and high‐level resistance to doravirine respectively.

Predicted high‐level resistance to doravirine (n = 601) was commonly caused by at least three DRMs (79.7%, n = 479), whereas intermediate resistance (n = 562) was most often caused by the presence of one or two DRMs (55.9%, n = 314). The DRM patterns among patients with at least three DRMs were extremely varied, and no link between the treatment group or DRM pattern was found.

To our knowledge, this is the first study looking at predicted doravirine resistance profiles in our setting. Doravirine follows a distinct resistance pathway compared to commonly used NNRTIs such as efavirenz and nevirapine [14].

In our ART‐naïve cohort, 8.3% of patients presented with at least one NNRTI DRM, but predicted resistance to doravirine (4.7%) remained significantly lower compared to efavirenz/ nevirapine resistance (9.4%). Studies in Europe showed lower doravirine resistance in ART‐naïve patients, with only 1.4% in France, Italy and Greece [22] and 1.8% in a Spanish study [23], likely due to less non‐B subtypes as well as differential treatment and monitoring protocols.

Although the predicted doravirine resistance profiles may look favourable in the ART‐naïve cohort, there are no clinical outcome data from settings without baseline genotyping. Real‐life data would be required to confirm these findings, as baseline genotyping is generally not available in LMICs. Only 15.2% of patients experiencing NNRTI‐based ART failure retained full predicted susceptibility to doravirine in our cohort. DRIVE‐BEYOND attempted to recruit patients with transmitted NNRTI mutations (K103N and G190A) and assess the efficacy of doravirine, but only 10 patients were recruited, and eight obtained virological suppression by week 48; one patient was lost to follow‐up at week 16 and one patient presented with virological failure at week 24 due to non‐adherence [24]. The DRIVE SHIFT study suggests that virologically suppressed patients can safely be switched to doravirine‐containing regimens, although the follow‐up period in this study was short (24 weeks) [25]. A small retrospective analysis of 16 patients who were switched to doravirine also suggests that patients with pre‐existing NNRTI resistance are able to maintain virological suppression for up to six months [26]. Of note, none of these patients experienced failure on NNRTI‐based regimens and only five of them presented with at least one NNRTI DRM at the time of switch. Also, the effect of key mutations, such as V106A/I/M, among others, has not been assessed. The uncertainty of the clinical impact of NNRTI mutations on doravirine efficacy is reflected in different resistance interpretation algorithms, where the Stanford HIVdb allocates a higher score to doravirine mutations, compared to ANRS and RIS algorithms [23].

The proportion of patients experiencing NNRTI‐based treatment failure and presenting with resistance to doravirine in our cohort (84.8%) was significantly higher compared to the proportion observed in two European studies (42.0% and 18.8%) [27, 28]. Although subtype C was underrepresented in both studies, Sterrantino *et al*. found subtype C accounted for the highest prevalence of doravirine resistance [28]. Some of these differences can be attributed to the high frequency of V106A/I/M (37.7%) in our cohort, compared to less than 3% in both European cohorts. Substitutions at position 106 have been identified as breakthrough mutations in *in vitro* experiments with doravirine in subtype A and C [14]. Specifically, the V106M mutation has been shown to be a signature mutation associated with efavirenz in subtype C virus in clinical settings [29]. Another factor contributing to the high prevalence of doravirine resistance in the South African cohort is the accumulation of NNRTI DRMs. Resistance to doravirine was caused by at least three DRMs in 79.7% of patients with high‐level resistance. Recent *in vitro* data suggest that the accumulation of NNRTI mutations increases doravirine resistance, despite the absence of doravirine‐specific mutations [30].

The findings from our NNRTI‐failure cohort suggest that routinely switching patients with existing NNRTI resistance to doravirine may not be advisable until we can confirm that virological suppression is greater in a clinical context than suggested from the resistance data.

These are observational data, and prone to a wide variety of potential biases, including referral bias. However, the demographic data reflect the overall South African ART cohort, which is predominantly female, and in an approximate age range. The ART‐naïve dataset used in this study is somewhat outdated. Moyo *et al*. [31] recently published the 2017 South African national household survey, where 22.1% of HIV‐infected, laboratory‐confirmed ART‐naïve participants presented with at least one NNRTI mutation. This is significantly higher than seen in our cohort; however, the household survey group was likely to include more defaulters, which might have influenced the NNRTI DRM prevalence. Moreover, we only looked at predicted resistance and not clinical outcomes when patients are initiated on or switched to doravirine‐based ART. Viral load suppression is possible in patients treated with first‐generation NNRTI‐based regimens, in the presence of mutations [32]. To date, no studies have been conducted to assess clinical outcomes in a head‐to‐head comparison between doravirine and dolutegravir‐based regimens.

## 4 Conclusions

Doravirine seems a suitable antiretroviral for evaluation in first‐line patients in our region who cannot tolerate or do not want to take dolutegravir‐based regimens, as both an initiation or switch option in virologically suppressed patients, with a predicted >90% efficacy. More real‐world data and/or clinical trial data are required to ascertain the clinical outcomes of doravirine in patients with pre‐existing NNRTI DRMs, especially from LMICs with circulating non‐B subtypes, before doravirine can safely be used to switch patients experiencing NNRTI‐based regimen failure.

Although doravirine is approved as initial therapy in patients who are ART‐naïve, it is currently recommended to obtain a genotype prior to the initiation of ART, which might not be feasible in many LMICs with large ART programmes. However, with the increased rollout of genotyping in some LMICs, baseline genotyping for a target population might be achievable. Alternatively, regular surveillance of pre‐treatment resistance, including doravirine, should be performed to inform the feasibility of using doravirine in first‐line treatment as has been done for first‐generation NNRTIs. On the other hand, the predicted resistance profiles in patients experiencing NNRTI‐based ART failure do not by itself substantiate the use of doravirine, until we have compelling data documenting viral suppression in the presence of these mutations.

## Competing interests

W.D.F.V is doing registration and investigator‐led studies that utilize doravirine, as well as several other antiretrovirals. M.M. started a full‐time role at ViiV Healthcare after the conceptualization and analysis of this study, but prior to publication. All others have no conflicts of interest to declare.

## Authors’ contributions

K.S. performed the research, analysed the data and wrote the paper. M.M. and W.D.F.V. conceptualized the study, interpreted the data and critically reviewed the paper. A.M.J.W and L.H. interpreted the data and critically reviewed the paper.

## Acknowledgements

None declared.

### Funding

No specific funding was received to perform this research.
